# Professor Allen J. Smith, M. D.

**Published:** 1903-07

**Authors:** 


PROFESSOR ALLEN J. SMITH, M. D., Dean of the Medical Department of the University of Texas, has resigned the Chair of Pathology in that school (effective August 1st, proximo) and has accepted the corresponding chair in the University of Pennsylvania, his *alma mater.* This is a great and serious loss to Texas. Professor Smith is almost idolized by his classes and he certainly brings to the position unusual proficiency and untiring zeal, and imparts to his lectures and laboratory experiments an interest that is as fascinating as it is instructive. We sincerely regret to lose him; but he is to be congratulated that his tender mother appreciates him so much that she must take him away from Texas to help her maintain her prestige and fame as one of the foremost educational institutions in the world. *Vale,* genial Smith. May every success, prosperity and happiness be yours always!

